# Pre-Consultation System Based on the Artificial Intelligence Has a Better Diagnostic Performance Than the Physicians in the Outpatient Department of Pediatrics

**DOI:** 10.3389/fmed.2021.695185

**Published:** 2021-11-08

**Authors:** Han Qian, Bin Dong, Jia-jun Yuan, Fan Yin, Zhao Wang, Hai-ning Wang, Han-song Wang, Dan Tian, Wei-hua Li, Bin Zhang, Lie-bin Zhao, Bo-tao Ning

**Affiliations:** ^1^Shanghai Engineering Research Center of Intelligence Pediatrics (SERCIP), Shanghai Children's Medical Center, School of Medicine, Shanghai Jiao Tong University, Shanghai, China; ^2^Department of Pediatric Intensive Care Unit, Shanghai Children's Medical Center, School of Medicine, Shanghai Jiao Tong University, Shanghai, China; ^3^Pediatric AI Clinical Application and Research Center, Shanghai Children's Medical Center, School of Medicine, Shanghai Jiao Tong University, Shanghai, China; ^4^Product Department, Hangzhou YITU Healthcare Technology Company, Hangzhou, China; ^5^Clinic Office of Outpatient, Shanghai Children's Medical Center, School of Medicine, Shanghai Jiao Tong University, Shanghai, China; ^6^Child Health Advocacy Institute, China Hospital Development Institute of Shanghai Jiao Tong University, Shanghai, China

**Keywords:** artificial intelligence, pre-consultation, outpatient, medical records, pediatric, electronic health record

## Abstract

Artificial intelligence (AI) has been deeply applied in the medical field and has shown broad application prospects. Pre-consultation system is an important supplement to the traditional face-to-face consultation. The combination of the AI and the pre-consultation system can help to raise the efficiency of the clinical work. However, it is still challenging for the AI to analyze and process the complicated electronic health record (EHR) data. Our pre-consultation system uses an automated natural language processing (NLP) system to communicate with the patients through the mobile terminals, applying the deep learning (DL) techniques to extract the symptomatic information, and finally outputs the structured electronic medical records. From November 2019 to May 2020, a total of 2,648 pediatric patients used our model to provide their medical history and get the primary diagnosis before visiting the physicians in the outpatient department of the Shanghai Children's Medical Center. Our task is to evaluate the ability of the AI and doctors to obtain the primary diagnosis and to analyze the effect of the consistency between the medical history described by our model and the physicians on the diagnostic performance. The results showed that if we do not consider whether the medical history recorded by the AI and doctors was consistent or not, our model performed worse compared to the physicians and had a lower average F1 score (0.825 vs. 0.912). However, when the chief complaint or the history of present illness described by the AI and doctors was consistent, our model had a higher average F1 score and was closer to the doctors. Finally, when the AI had the same diagnostic conditions with doctors, our model achieved a higher average F1 score (0.931) compared to the physicians (0.92). This study demonstrated that our model could obtain a more structured medical history and had a good diagnostic logic, which would help to improve the diagnostic accuracy of the outpatient doctors and reduce the misdiagnosis and missed diagnosis. But, our model still needs a good deal of training to obtain more accurate symptomatic information.

## Introduction

Artificial intelligence (AI) has been deeply applied in the medical field and has shown broad application prospects. AI has been focusing on the imaging diagnosis for a long time. For example, in the terms of iconography (1, 2) and pathology (3–5) diagnosis, the diagnostic efficiency of the AI even exceeds compared to the most experienced doctors, effectively improving the efficiency and accuracy of the medical staff. With the continuous development of the deep learning (DL) technology, the application scenarios of the AI continue to expand at the same time. Currently, AI has been able to diagnose common diseases, evaluate anesthesia, and manage pharmacies (6–8).

With the development of the medical technology, more and more diversified methods of observing diseases have made medical information more complex and the clinical decision-making also more cumbersome. To make a comprehensive decision, the doctors usually need to evaluate large amounts of the clinical information. Among them, the electronic health record (EHR), as an enormous electronic data repository, represents a wide variety of the clinical information. AI has gradually become a powerful tool for mining EHR data to assist human doctors in the clinical decision-making. For example, the application of the AI in the EHR has been effectively developed and it has been used to enhance the surgical decision-making (9), healthcare (10), outcome prediction (11), heart failure prediction (12), and suicide risk stratification (13).

In the process of the outpatient consultation, in order to formulate a diagnosis for any visiting patient, the doctors often use the hypothetical coding reasoning (14). Starting from the chief complaint, the doctor then asks the targeted and appropriate questions related to the chief complaint and forms an initial small feature dataset based on the answers of the patient. In turn, the doctor will form a differential diagnosis and decide which features to obtain next to rule out the differential diagnosis. The most useful features are identified one after another. After a continuous process of “reasoning-diagnosis-rereasoning-rediagnosis,” when the probability of a certain diagnosis reaches a predetermined acceptable level, the process stops and the diagnosis is output. In this way, an acceptable possibility of the diagnosis can be achieved with only a few features, without having to deal with the entire feature of the dataset. Liang et al. proposed a data mining framework for the EHR data, which was trained and validated by analyzing 101.6 million data points from 1,362,559 pediatric patients. The model demonstrated the high diagnostic accuracy across the multiple organ systems (14).

We designed the pre-consultation system based on the AI, of which the core algorithm is similar with the data mining framework proposed by Liang et al. (14). It applies the automated natural language processing (NLP) system to communicate with the patients and uses DL techniques to extract the symptomatic information. It can mimic the “reasoning-diagnosis” process of the physicians to get the primary diagnosis and finally outputs structured EHRs. Pre-consultation system is an important supplement to the traditional face-to-face consultation, which refers that people could describe their conditions in the form of answering questions on the mobile terminal through the AI pre-consultation system and could obtain the preliminary diagnosis and medical advice before they visit a doctor.

However, it is still challenging for the AI to analyze and feedback complex text data, finding expression in the vast quantity of data, high dimensionality, and data sparsity in the medical data (15). The AI pre-consultation system should be designed to have the ability to extract the clinical information from the free text with a high precision and recall ratio and the ability to make a preliminary diagnosis. Therefore, a reasonable assessment of the data capture, learning ability, and preliminary diagnosis level is the key area of the research and development of the AI pre-consultation system. In order to understand the interrogation capabilities and preliminary diagnosis level of the commercial AI pre-consultation system, we collected a total of 5,296 medical records of 2,648 patients who used this system in the pediatric outpatient department of our hospital. Each patient has two medical records, one is generated by the pre-consultation system, and the other is from the outpatient physicians. We comprehensively evaluated the performance of the pre-consultation system and compared the internal logical differences between the AI and human physicians, with the hope to provide the methods and references for the follow-up-related clinical application scenarios.

## Methods

### Data Collection

This study included 2,648 pediatric patients who used our AI pre-consultation system before the traditional routine outpatient visits in the Shanghai Children's Medical Center (SCMC), Shanghai Jiao Tong University School of Medicine from November 2019 to May 2020. Each patient has two medical records, with one collected by our AI pre-consultation system, and another collected by the outpatient doctors during the traditional face-to-face consultation, both including information such as payment account number, registration date, treatment department, doctor level, age, gender, chief complaint, current medical history, and preliminary diagnosis. This study was approved by the Ethics Committee of SCMC (SCMCIRB-K2019020-2). All the included recorded cases signed a written informed consent and the recorded private data were deleted or obscured.

### Design of the Artificial Intelligence Pre-Consultation System

This pre-consultation system combining the AI and the EHR is jointly developed by the SCMC and the Yitu Technology Company. The model applies NLP system, synonymous word database, medical knowledge graph technology, etc., that can standardize the free text input by the patients and extract the feature values from it. Then, through the “question-answer” system which was constructed after model training, the negative symptoms and the negative symptoms are sequentially obtained and a structured symptom description is output. It applies DL technology and NLP system and can imitate the diagnostic logic of the doctor for the reasoning and deduction. Based on DL technology, the pre-consultation system can imitate the reasoning logic of the doctor and get a preliminary diagnosis based on the acquired disease information. We selected 59,041 high-quality EHRs manually labeled by the professional doctors and informatics experts and trained the model by using the XGBoost algorithm. The core algorithm of the model is similar to the model proposed by Liang et al. (14), but our model has been updated and iterated based on the data of the information system of our hospital. The AI pre-consultation system uses NLP technology, combining synonyms database, and medical knowledge graph technology, etc., to carry out the structured processing, extract the feature values, and obtain the structured symptomatic description.

### Data Processing

**Figure 1:**
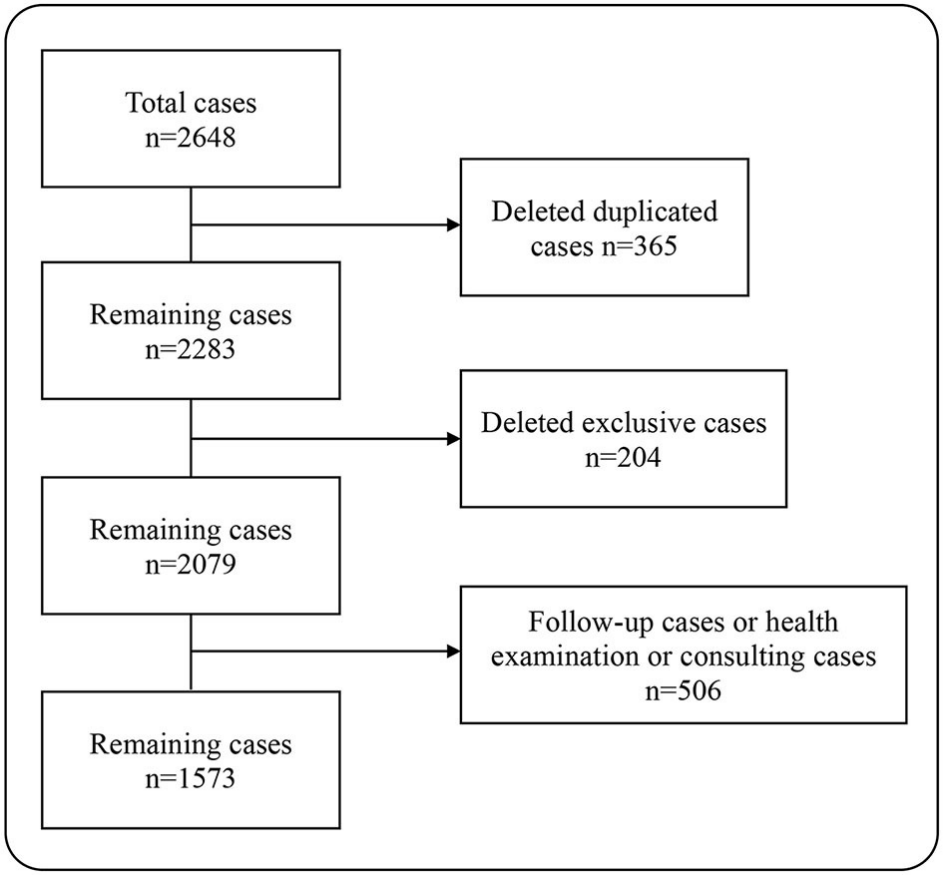
Data processing.

In this study, a total of 2,648 pairs of pediatric outpatient records were included. After deleting the duplicate records, there were remaining 2,283 pairs of medical records. Then, we excluded cases that could not be matched, i.e., the cases that only used the pre-consultation system on the mobile terminal, but not registered at the outpatient clinic. At this point, there were 2,079 pairs of the medical records left. Some patients went to the hospital just for the health consultation, physical examination, and medicine purchase, so they did not describe their conditions seriously to the AI, leading to the deficiency of the information extracted by the AI whether in quantity or in quality. For the sake of fairness, we deleted those records, a total of 506 pairs. At last, 1,573 pairs of the outpatient EHRs were included in the analysis, containing 31,460 data points (Figure 1).

### Scoring Rules and Dataset Definition

To analyze whether the medical history consistency between the AI and physicians influences the diagnostic performance, we screened the records based on whether the medical history, including the chief complaint and the history of present illness (HPI), collected by the AI and the doctors is consistent and formed five datasets. The following is our definition of these five datasets. Dataset one contains all the 1,573 pairs of medical records included in the analysis. Dataset two contains a total of 935 pairs of medical records in which the chief complaint described by the AI is consistent with the physicians. Dataset three contains a total of 742 pairs of medical records in which the HPI described by the AI is consistent with the physicians. Dataset four contains a total of 536 pairs of medical records in which the chief complaint and the HPI described, respectively, by the AI and the physicians are both consistent. Dataset five contains all the medical records in which both the chief complaint and the HPI described by the AI and the doctors are inconsistent.

**Figure 2:**
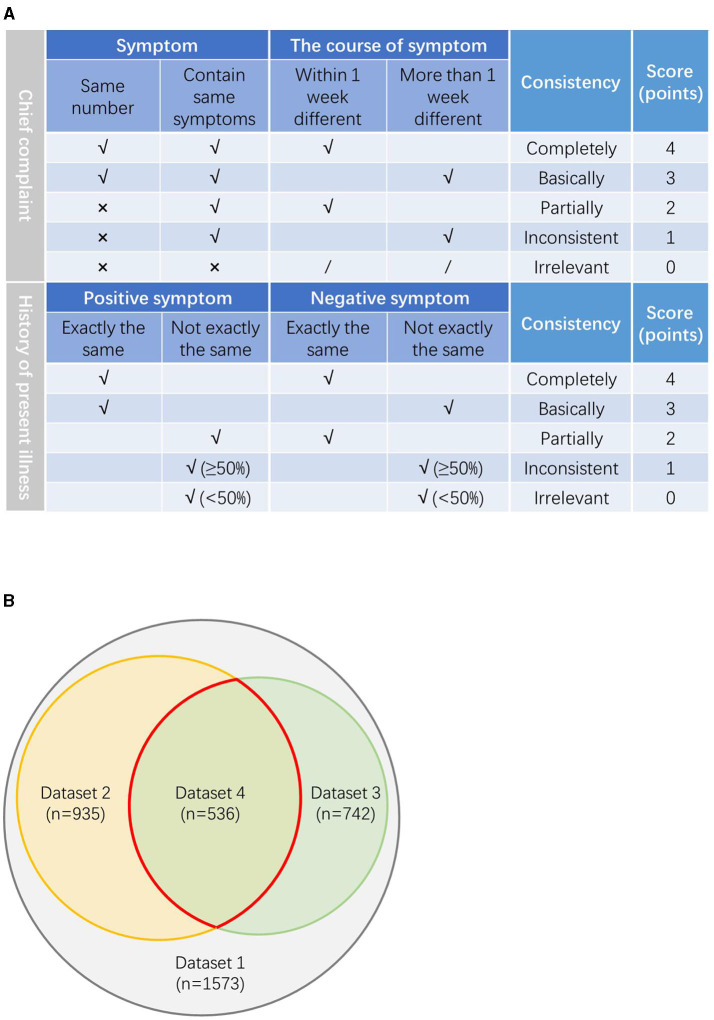
**(A)** The evaluation rules on the consistency between the chief complaint and the history of present illness (HPI) described by our model and physicians. **(B)** The relationship between the datasets.

The scoring system was determined by several senior doctors after thorough consideration and discussion. We invited the three senior doctors to evaluate the consistency of the chief complaint and the HPI described by the AI and the physicians, respectively. Each expert first scored the medical records independently and then divided the dataset based on the average scores of the three experts. The evaluation rules are as follows and are shown in Figure 2A.

For the chief complaint, if the symptoms of the chief complaint are exactly the same and the difference of the symptom course is within 7 days, it is defined as completely consistent, score 4 points; if the main complaint symptoms are exactly the same, but the course of the symptom is more than 1 week different, it is defined as basically consistent, score 3 points; if the number of the main complaint symptoms is different, but there are same symptoms with the course >1 week apart, it is defined as partially consistent, score 2 points; if the number of the main complaint symptoms is different, but there were same symptoms with the course differed by more than a week, it is defined as inconsistent, score 1 point; if the number of the main complaint symptoms is different and there are not same symptoms, it is defined as irrelevant, score 0 point.

For the HPI, if the positive symptoms and the negative symptoms are exactly the same, it is defined as completely consistent, score 4 points; if the positive symptoms are completely the same, but the negative symptoms are not completely the same, it is defined as basically consistent, score 3 points; if the positive symptoms are not completely the same, but the negative symptoms are exactly the same, it is defined as partial consistent, score 2 points; if both the positive symptoms and the negative symptoms are not exactly the same, but the number of the same symptoms is more than half, it is defined as inconsistent, score 1 point; if both the positive symptoms and the negative symptoms are not exactly the same and the number of the same symptom is less than half, it is defined as irrelevant, score 0 point.

Three doctors evaluated the consistency of the medical records according to the above rules and scores. About five datasets were produced according to the average scores. Dataset two includes all the cases with average score ≥ 3 points in the consistency evaluation of the chief complaint; dataset three includes all the cases with average score ≥ 3 points in the consistency evaluation of the HPI; dataset four includes all the cases with average score ≥ 3 points both in the consistency evaluation of the chief complaint and the HPI. Dataset five includes all the remaining cases with average score < 3 points both in the consistency evaluation of the chief complaint and the HPI. The relationship between the datasets is shown in Figure 2B.

### Flow of a Visit of the Patient

When a child has symptoms, parents can enter the main symptoms or medical appeals through the typing or smart voice on the mobile phone in advance when they plan to make an appointment with the doctor. Our model could automatically extract the key information and conduct further asking according to the medical logic to complete the inquiries about the symptoms, past history, allergy history, inferred symptoms, and other medical information. The system will predict the disease of the patient through an algorithm model based on the medical records of the patient and give a preliminary diagnosis. Thus, the system will regenerate the structured data according to the writing standards and organize them into standardized outpatient electronic medical records, which can be directly cited by the outpatient doctors. Of course, the doctors were not allowed access to the diagnosis of the AI. The patients can also check their medical records written by the AI on their phones.

**Figure 3:**
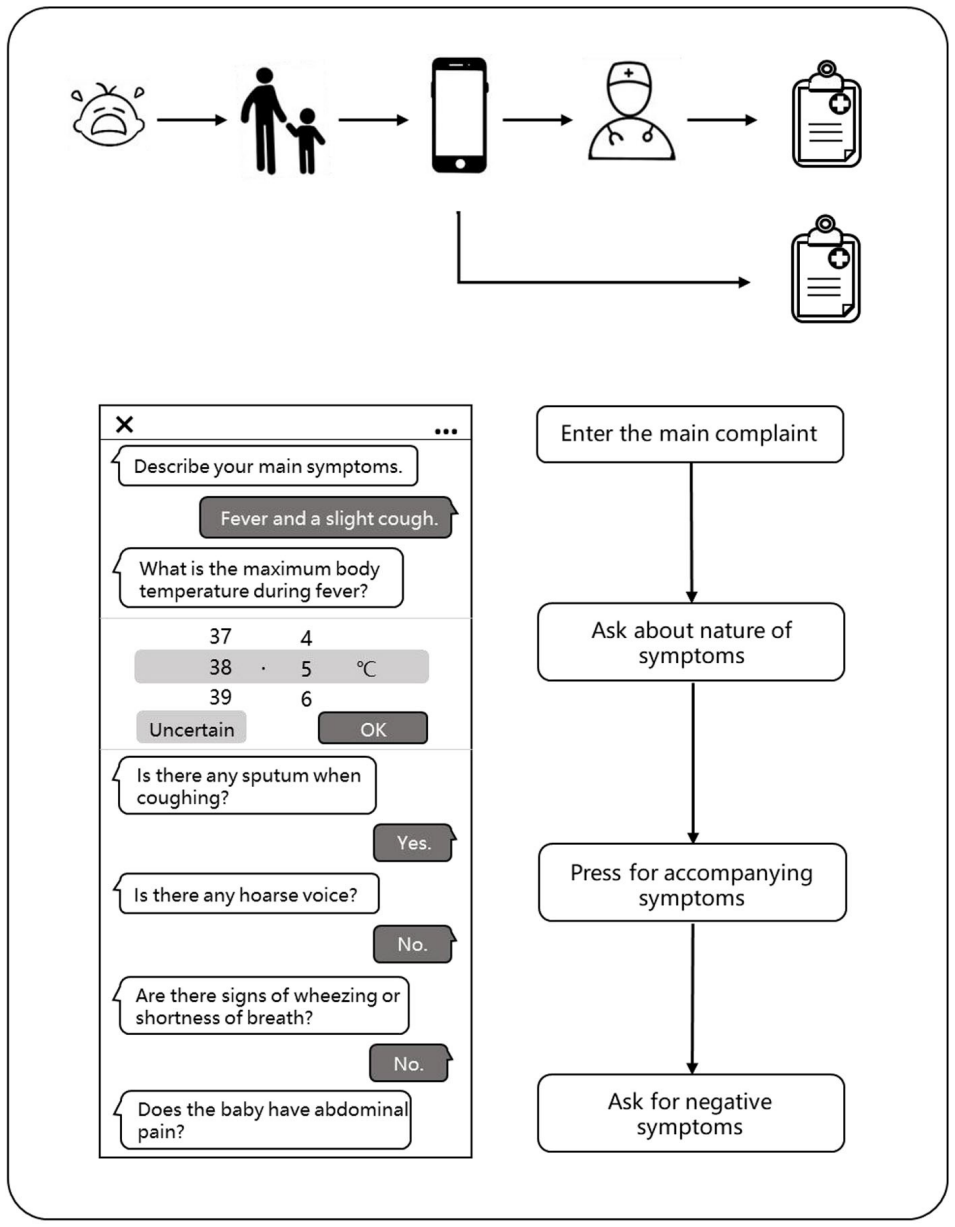
The flow of a visit of the patient.

After the patients complete the appointment registration, they begin their traditional face-to-face consultation to the outpatient physicians. The physicians can choose to refer to or not refer to the medical records (except the diagnosis results) obtained by the AI pre-consultation system and obtain the conditions of the patients through asking questions, physical examination, and laboratory reports. Finally, the physicians would give their diagnosis opinion according to the information they mastered and also generate a brand new electronic medical record (Figure 3).

### Statistical Analysis

We used the statistical software Statistical Package for Social Sciences (SPSS) Version 22.0. Armonk, NY: IBM Corp. to figure out all the statistics. In this study, the age of the children is a continuous variable, but does not follow a normal distribution, so we use the median (interquartile range) to describe the age variable. The other variables are the enumeration data and are described with frequency (ratio). In addition, we used F1 score to evaluate the diagnostic performance of the AI pre-consultation system and the physicians. F1 score is used as a statistical measure to evaluate the performance of the classifiers, which is the harmonic average of the precision and recall. Precision refers to the percentage of the true positive samples among the samples judged to be positive by the classifier. The recall rate refers to the percentage of the positive samples judged by the classifier to the total positive samples. The value of F1 score ranges from 0 to 1. The larger the F1 score, the better the performance of the classifier.

## Results

### Basic Information

The initial dataset contains the EHRs of 2,648 outpatients. Every patient owned the two EHRs, which were recorded by the pre-consultation system and outpatient doctor, respectively. Every EHR contained the medical information such as medical department, chief complaint, HPI, and preliminary diagnosis. We deleted duplicate cases, unmatched cases, follow-up cases, consulting cases, and physical examination cases, eventually included 1,573 cases into the analysis. The median age of the enrolled patients was 3.3 years (interquartile range, 1.2–6.0) including 245 males (15.58%) and 1,328 females (84.42%). Among all the patients, 62.17% patients had respiratory diseases, 30.64% patients had digestive system diseases, and 3.05% patients had urinary tract diseases.

### Diagnostic Performance of the Artificial Intelligence in the Dataset One is Worse Than the Doctors

We evaluated the most frequently occurring diseases with F1 score to evaluate the diagnostic performance of the AI and the physicians including upper respiratory tract infection (URTI), bronchitis, upper airway cough syndrome (UACS), gastroenteritis, mesenteric lymphadenitis (ML), and urinary tract infection (UTI). We found that the AI achieved a lower average F1 score compared to the doctors (0.825 vs. 0.912) with a poor diagnostic performance in the above diseases including URTI (0.810 vs. 0.906), bronchitis (0.755 vs. 0.834), UACS (0.766 vs. 0.870), gastroenteritis (0.864 vs. 0.966), ML (0.872 vs. 0.950), and UTI (0.879 vs. 0.947) (Table 1).

**Table 1:** Diagnostic performance of the AI model and the physician for the dataset one (*n* = 1,573).

**Disease**	**AI (F1 score)**	**Physician (F1 score)**
URTI	0.810	0.906
Bronchitis	0.755	0.834
UACS	0.766	0.870
Gastroenteritis	0.864	0.966
ML	0.872	0.950
UTI	0.879	0.947
Average	0.825	0.912

*AI, artificial intelligence; URTI, upper respiratory tract infection; UACS, upper airway cough syndrome; ML, mesenteric lymphadenitis; UTI, urinary tract infection*.

### Diagnostic Performance of the Artificial Intelligence in the Dataset Two and the Dataset Three is Worse Than the Doctors but Better Compared to the Dataset One

To analyze the impact of the different medical records on the diagnostic performance between the AI and the doctors, we evaluated the EHRs with the consistent chief complaint or the HPI. For dataset two, the AI showed a poor diagnostic performance. The F1 scores in the AI were lower compared to the physicians including URTI (0.859 vs. 0.907), bronchitis (0.789 vs. 0.831), UACS (0.844 vs. 0.897), gastroenteritis (0.918 vs. 0.972), ML (0.910 vs. 0.950), and UTI (0.929 vs. 0.949). The F1 scores of the AI have increased significantly compared to the dataset one. For dataset three, we found similar results. The average F1 score of the AI was still lower compared to the doctors (0.886 vs. 0.921), but higher compared to the dataset one (Tables 2, 3).

**Table 2:** Diagnostic performance of the AI model and the physician for the dataset two (*n* = 935).

**Disease**	**AI (F1 score)**	**Physician (F1 score)**
URTI	0.859	0.907
Bronchitis	0.789	0.831
UACS	0.844	0.897
Gastroenteritis	0.918	0.972
ML	0.910	0.950
UTI	0.929	0.949
Average	0.875	0.918

*AI, artificial intelligence; URTI, upper respiratory tract infection; UACS, upper airway cough syndrome; ML, mesenteric lymphadenitis; UTI, urinary tract infection*.

**Table 3:** Diagnostic performance of the AI model and the physician for the dataset three (*n* = 742).

**Disease**	**AI (F1 score)**	**Physician (F1 score)**
URTI	0.868	0.904
Bronchitis	0.783	0.838
UACS	0.910	0.912
Gastroenteritis	0.931	0.968
ML	0.926	0.956
UTI	0.899	0.948
Average	0.886	0.921

*AI, artificial intelligence; URTI, upper respiratory tract infection; UACS, upper airway cough syndrome; ML, mesenteric lymphadenitis; UTI, urinary tract infection*.

### Diagnostic Performance of the Artificial Intelligence in the Dataset Four is Better Than the Doctors

Subsequently, we evaluated the EHRs with the consistent chief complaint and the HPI (dataset four). We found that the diagnostic efficiency of the AI had greatly improved and even surpassed compared to the doctors. AI not only had an average F1 score higher compared to the doctors (0.931 vs. 0.920), but also achieved higher F1 scores in URTI (0.906 vs. 0.899), bronchitis (0.828 vs. 0.833), UACS (0.942 vs. 0.913), gastroenteritis (0.973 vs. 0.970), ML (0.976 vs. 0.958), and UTI (0.962 vs. 0.946) compared to the doctors (Table 4).

**Table 4:** Diagnostic performance of the AI model and the physician for the dataset four (*n* = 536).

**Disease**	**AI (F1 score)**	**Physician (F1 score)**
URTI	0.906	0.899
Bronchitis	0.828	0.833
UACS	0.942	0.913
Gastroenteritis	0.973	0.970
ML	0.976	0.958
UTI	0.962	0.946
Average	0.931	0.920

*AI, artificial intelligence; URTI, upper respiratory tract infection; UACS, upper airway cough syndrome; ML, mesenteric lymphadenitis; UTI, urinary tract infection*.

## Discussion

This is an era of the rapid development of the AI technology. AI is able to perform the abstract analysis on the complex data to simulate the human learning behavior and continuously improve its own performance (16). AI has been successfully applied to various medical scenarios such as virtual assistants, medical imaging, auxiliary diagnosis, and drug development (17). However, due to the difficulties of the complex data extraction, text conversion, and association analysis, the application of the AI in the EHR analysis hits a bottleneck (18, 19). We developed the AI pre-consultation system based on the DL and NLP. In other words, the pre-consultation system is a form of the AI processing the EHR data. In this study, we compared the diagnostic performance of the AI and the doctors and analyzed the probable factors that affect the processing of the AI of the EHR data. We found that the diagnostic efficiency of the AI was better compared to the doctors based on the standardized EHR data (which means the AI has the same diagnostic condition with doctor) and AI could assist the doctor to make a clinical diagnosis.

In the beginning, when we used the primary data (dataset one), namely the 1,573 pairs of the untreated EHRs, to analyze the diagnostic performance of the AI and the doctors, we found that the performance of our model was far from satisfactory and was inferior to that of the doctors. However, this finding is different from the finding proposed by Liang et al. (14), who found that the AI was comparable to the experienced pediatricians in diagnosing the common pediatric diseases. Why do we get different results when the core algorithm for the model is similar? We speculated that it is the quality of the medical records dataset used to diagnose that influences the diagnostic performance of the model. In this study, both the training model and the validation model used standardized medical records. In other words, these medical records were in advance manually annotated by the senior attending physicians with more than 25 years of clinical practice experience, so that the data were described in a harmonized manner. But, we did not further process the medical records. In fact, for some reasons, the medical records obtained by the AI and the doctors for the same patient may not be exactly the same or even irrelevant. This means that the AI and the doctors were not compared under the same conditions, which are the most obvious reason for the low diagnostic efficiency of our model for dataset one. During the pre-consultation system, some medical terms may be too obscure for the patient to understand, so that they might provide wrong information to the AI. Besides, some parents may have not used the pre-questioning system seriously, which leads to the information obtained by the AI was not accurate or complete (20, 21). Just like a chain reaction, if the AI does not get the correct answer to a question, it will later acquire the wrong features and make a wrong diagnosis. In addition, the missing data may cause the AI algorithm to use only the remaining data for reasoning, leading to an increase in the error rates (22, 23).

Gianfrancesco et al. (24) believed that the bias in processing the EHR data may cause the AI to make incorrect decisions. In this study, the chief complaint and the HPI recorded by the AI in some EHRs were different compared to the doctors, which could cause the information bias. Therefore, we screened out the EHRs with the consistent chief complaints (dataset two, *n* = 935) and the medical records with the consistent HPI (dataset three, *n* = 742). As a result, the diagnostic level of the AI had improved significantly, but it was still worse compared to the doctors. AI is usually designed to establish a relationship between the diseases and all the information it obtained, including all the symptoms, physical examination, and test results, and to provide a comprehensive and broad diagnosis (25, 26). Hence, this design often results in a high false-positive rate. In contrast, the doctors would make a more targeted diagnosis based on their own experience. Thus, it can be seen that the AI rarely misses the diagnosis, but doctors miss the diagnosis. Furthermore, we screened the EHRs with the consistent chief complaint and the consistent HPI and found that the average F1 score of the AI has further improved, even exceeding the doctors. This shows that the performance of our model is not inferior compared to the doctors based on the same diagnosis situations and the logic behind this AI pre-consultation system is feasible and the application of the AI to the auxiliary diagnosis of the diseases might improve the efficiency of the outpatient clinics.

The pre-consultation system will be conductive to improve the diagnostic accuracy of the outpatient doctors and reduce the occurrence of the misdiagnosis and missed diagnosis. It is an important auxiliary tool for the outpatient doctors in the daily face-to-face consultations and has the certain promotion value and application significance in the specific scenarios. On one hand, due to the huge number of patients in the outpatient clinics of the regional or national medical centers, the doctors often overwork and short visits for the individual patients, which lead to the inevitable misdiagnosis and missed diagnosis. Our model can obtain the structured EHRs and is designed to output the three diagnosis results, so it has a high sensitivity. Therefore, the diagnosis results output by the model have an important prompting function for human doctors. On the other hand, the medical level varies among the regions, which is more obvious in pediatrics. Therefore, the promotion of our AI pre-consultation system will help to shorten the medical gap between the regions.

The previous analysis has proved that when the chief complaint and the HPI described by the AI and the doctors were both consistent, the diagnostic efficacy of the AI was not second compared to the doctors. It is worth noting that the information of the patient obtained through the AI pre-consultation system in the form of the questionnaires is more comprehensive including all the probable positive symptoms and the negative symptoms. In comparison to the outpatient consultation, the data obtained by the AI and the medical records written by the AI are more structured, which are conducive to the differential diagnosis of the outpatient physicians. Of course, the shortcoming of our model is also obvious: the ability to accurately collect the medical history needs to be improved. In comparison to the image data, the AI faces more complicated situations in the EHR processing. For the pre-consultation system, the biggest challenge is to improve the accuracy of the initial dataset. This can be improved by reducing the technical terms and adding the noun explanations (in the form of text, picture, or video) in the interaction interface between the patients and the AI. In addition, the consistency of the diagnostic capabilities of the AI among the various disease systems is also very important. The number of the guidelines and materials for the different diseases that the machine masters during practice is different, which leads to the differences in its diagnostic capabilities in the different systems. But, we believe that this limitation will also be overcome with the advancement of medical and health services. In addition, optimizing the internal logic of the AI and developing algorithms based on the biased data will also help to raise the diagnostic accuracy.

In summary, the combination of the AI and the EHR system has broad application prospects. The AI pre-consultation system will contribute to raise the diagnostic accuracy in the outpatient clinics and reduce the incidence of the misdiagnosis and missed diagnosis, which make it an important auxiliary tool for the outpatient doctors in the daily visits. Therefore, the promotion of the AI pre-consultation system will help to shorten the medical gap between the regions and promote the realization of the ideal of common health for the people.

## Data Availability Statement

The original contributions presented in the study are included in the article/supplementary material, further inquiries can be directed to the corresponding author/s.

## Author Contributions

HQ, BD, J-jY, ZW, H-nW, B-tN, and L-bZ contributed to the conception and design of the study. L-bZ and B-tN contributed to the administrative support. L-bZ contributed to the provision of the study materials or the patients. HQ, FY, ZW, H-nW, BD, J-jY, H-sW, DT, W-hL, and B-tN contributed to the collection and assembly of the data. HQ, BD, FY, B-tN, and L-bZ contributed to the analysis and interpretation of the data. All the authors contributed to the writing and final approval of the manuscript.

## Funding

The study was supported by the Clinical Science and Technology Innovation Program of Shanghai Shenkang Hospital Development Center (SHDC12020605), Science and Technology Innovation — Biomedical Supporting Program of Shanghai Science and Technology Committee (19441904400), and Program for Artificial Intelligence Innovation and Development of Shanghai Municipal Commission of Economy and Informatization (2020-RGZN-02048).

## Conflict of Interest

The authors declare that the research was conducted in the absence of any commercial or financial relationships that could be construed as a potential conflict of interest.

## Publisher's Note

All claims expressed in this article are solely those of the authors and do not necessarily represent those of their affiliated organizations, or those of the publisher, the editors and the reviewers. Any product that may be evaluated in this article, or claim that may be made by its manufacturer, is not guaranteed or endorsed by the publisher.
